# A mechanism for 1,4-Benzoquinone-induced genotoxicity

**DOI:** 10.18632/oncotarget.10184

**Published:** 2016-06-20

**Authors:** Mi Young Son, Chu-Xia Deng, Jan H. Hoeijmarkers, Vivienne I. Rebel, Paul Hasty

**Affiliations:** ^1^ Department of Molecular Medicine and Institute of Biotechnology, University of Texas Health Science Center at San Antonio, San Antonio, Texas, USA; ^2^ Faculty of Health Sciences, University of Macau, Macau SAR China; ^3^ Department of Genetics, Cancer Genomics Netherlands, Erasmus MC, The Netherlands; ^4^ Department of Cellular and Structural Biology, University of Texas Health Science Center at San Antonio, San Antonio, Texas, USA; ^5^ The Cancer Therapy Research Center, University of Texas Health Science Center at San Antonio, San Antonio, Texas, USA; ^6^ The Barshop Center of Aging, University of Texas Health Science Center at San Antonio, San Antonio, Texas, USA; ^7^ Greehey Children's Cancer Research Center, University of Texas Health Science Center at San Antonio, San Antonio, Texas, USA; ^8^ Current address: BioAffinity, San Antonio, Texas, USA

**Keywords:** Fanconi anemia, double strand break repair, replication fork maintenance, type 1 topoisomerase

## Abstract

Benzene is a common environmental toxin and its metabolite, 1-4-Benzoquinone (BQ) causes hematopoietic cancers like myelodysplastic syndrome (MDS) and acute myeloid leukemia (AML). BQ has not been comprehensively assessed for its impact on genome maintenance, limiting our understanding of the true health risks associated with benzene exposure and our ability to identify people with increased sensitivity to this genotoxin. Here we analyze the impact BQ exposure has on wild type and DNA repair-defective mouse embryonic stem (ES) cells and wild type human cells. We find that double strand break (DSB) repair and replication fork maintenance pathways including homologous recombination (HR) and Fanconi anemia (FA) suppress BQ toxicity. BQ-induced damage efficiently stalls replication forks, yet poorly induces ATR/DNA-PK_CS_ responses. Furthermore, the pattern of BQ-induced γH2AX and 53BP1foci is consistent with the formation of poly(ADP-ribose) polymerase 1 (PARP1)-stabilized regressed replication forks. At a biochemical level, BQ inhibited topoisomerase 1 (topo1)-mediated DNA ligation and nicking *in vitro*; thus providing mechanism for the cellular phenotype. These data are consistent with a model that proposes BQ interferes with type I topoisomerase's ability to maintain replication fork restart and progression leading to chromosomal instability that has the potential to cause hematopoietic cancers like MDS and AML.

## INTRODUCTION

Few occupational or environmental hazards are unambiguously linked to the development of myeloid neoplasms. This is partly due to the uncertainty between the time of exposure and the appearance of symptoms. In addition, criteria for diagnosing these diseases have dramatically changed over the years, in particular for myelodysplastic syndrome (MDS), which complicates the evaluation of old patient records. Nevertheless, decades of follow-up studies and reexamining pathology reports show that adults who were exposed at the work place to cumulative high levels, or chronic low levels of benzene have an increased risk of developing MDS or acute myeloid leukemia (AML), respectively [1–3].

Benzene is a colorless volatile liquid hydrocarbon found in coal tar and petroleum and is used to make numerous chemical products including detergents, insecticides and motor fuels [4, 5]. The primary benzene metabolite to cause genomic damage is 1,4-benzoquinone (BQ) and is believed to be responsible for the myelotoxicity/myeloid neoplasms observed in the bone marrow of people who have been exposed to increased levels of benzene [4]. In addition to specific industry-related work places, benzene concentrations are also significantly higher in and around cities with a high coal or oil-based energy consumption, as well as in rural areas subject to heavy pesticide use. This raises the question whether environmental benzene pollution can be a contributing factor to MDS and AML development. In support of this notion is the increased incidence of childhood leukemia observed in Harris county, Texas (Houston area), which houses several petroleum and chemical industries that are associated with air pollutants, including benzene [6]. In addition, several studies report that MDS is diagnosed on average a decade earlier in Asian countries compared to Western countries [7–11]. Since the former countries use mostly coal to fuel their industries [12] and households, systemic exposure to environmental toxins such as benzene, could be responsible for their earlier onset of MDS.

An alternative explanation for the difference in age of MDS onset between Western and Asian populations is a difference in genetic make-up. There is no doubt that an individual's machinery responsible for proper genome maintenance suppresses hematologic cancers [13]. This is particularly apparent in diseases characterized by poor DNA repair capacity [14, 15]. For example, patients with Fanconi Anemia (FA) are defective for genome maintenance and exhibit a high incidence of MDS and AML [16]. Moreover, alternative SNPs in multiple DNA repair genes were found to associate with hematotoxicity in adults routinely exposed to benzene [17]. The difference in childhood leukemia onset in specific Texas counties may also have an underlying genetic basis. Texas is home to a large Hispanic population and several studies have shown that Hispanic children exhibit a significantly higher incidence and worse outcome of acute lymphocytic leukemia (ALL) compared to non-Hispanic children. Specifically, Hispanic children in Texas who developed ALL overrepresented several polymorphisms in genes known to associate with cancer development when mutated [18, 19]. This same group of children exhibited a significantly higher risk for developing secondary MDS/AML after receiving etoposide (a topoisomerase type II inhibitor that was part of their ALL treatment) [20]. Thus, occupational and environmental exposures to benzene, as well as poor DNA damage response/repair can enhance the risk for the development of hematologic cancers like MDS and AML.

Very little is known about the mechanisms required to cope with benzene-induced DNA damage. This lack of understanding hampers a more detailed assessment of the risk benzene exposure poses to people and our ability to identify those at high risk for MDS and AML. The current study was initiated to increase our understanding of the consequences of benzene-induced DNA damage and the mechanisms required to repair it. We found that the benzene metabolite, BQ induced chromosomal breaks and rearrangements as well as stalled replication forks, which required DSB repair and the FA pathway to correct. Furthermore, BQ directly interfered with the ability of type 1 topoisomerase (topo 1) to nick DNA and relieve supercoiling. Topo 1 interference is consistent with the observations that BQ-induced damage causes replication fork regression that could lead to chromosomal breaks and rearrangements, especially if DSB repair and FA pathways are compromised. Thus, these data support the observations that benzene enhances risk of MDS and AML especially for those with compromised genome maintenance capacity.

## RESULTS

### Cells defective for DSB repair and replication fork stability are hypersensitive to BQ

We previously developed a screening system to identify the DNA repair pathway(s) most important for repairing DNA lesions induced by a given genotoxin [21]. This screen will generate a genotoxic profile of the toxin under investigation and it is based on a comprehensive set of mouse embryonic stem (ES) cells defective for specific DNA repair pathways, including those that repair base lesions, replication errors, double strand breaks (DSBs) and interstrand and intrastrand crosslinks (Table 1) [21]. To create a BQ genotoxic profile, we performed a dose response curve for each mutant cell line to determine the threshold BQ concentrations that reduce cell survival [22]. Mutant cells that are more sensitive to threshold BQ concentrations as compared to their parental control reveal a pathway important for correcting BQ-induced damage. Thus, this screening method takes an unbiased approach to discover the DNA repair pathways most important for correcting BQ-induced damage.

**Table 1:** Summary of mutant ES cells

Control cells	Gene	Mutations	Function
AB1.1	Msh2	−/−	MMR
AB2.2	Brca2	Exon 27 deletion	HR
	Blm	88% decrease	Helicase/HR
	Recql5	−/−	Helicase/HR
	Trex2	−/−	Exonuclease/RF
	FancB	Exon 2 deletion	ICLR/RF
B44	Xpa	−/−	NER
	Xpc	−/−	NER
J1	Ku70	−/−	cNHEJ
TC1	H2AX	−/−	DDR/HR
	Brca1	BRCT deletion	DDR/HR/NHEJ
IB10	Rad18	−/−	Lesion bypass
E14 (IB10)	Rad52	−/−	HR
	Rad54	−/−	HR
	Mus81	−/−	Endonuclease/HR
	Ercc1	−/−	NER/HR/ICLP

Cells were used that were ablated for nucleotide excision repair (NER) (*Xpa* [73], *Xpc* [74]), mismatch repair (MMR, *Msh2*) [75], lesion bypass (*Rad18*) [76], the Fanconi anemia (FA) pathway (*Fancb*) [77], nonhomologous end joining (NHEJ, *Ku70*) [78]. Complete ablation of homologous recombination (HR) is cell lethal [79]; therefore, null cells were used for genes that contribute to, but are not essential for HR (*H2ax* [80], *Rad52* [81], *Rad54* [82]). Cells were used that are partially defective for essential proteins that include a deletion of *Brca2* exon 27 [26] and deletion of *Brca1* exon 11 [83]. Cells were used that are defective for HR regulation that include mutations in the helicases *Blm* [84] and *Recql5* [85]. Cells were used that are defective for endonucleases (*Mus81* [86] and *Ercc1* [87]) that can be used during HR and interstrand crosslink repair (ICLR) and exonucleases (*Trex2*) [88] that can be used for lesion bypass.

**Figure 1:**
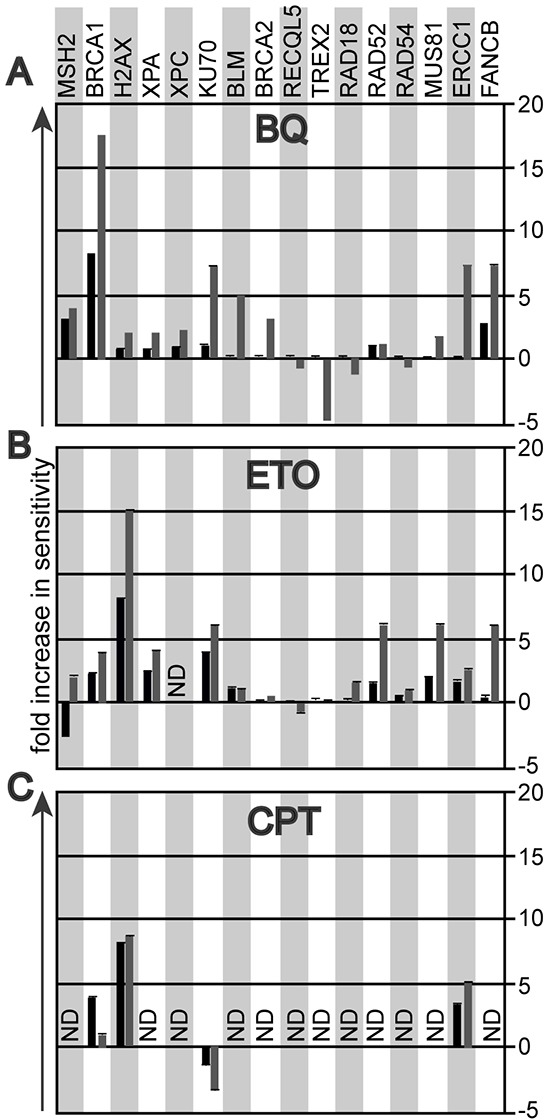
The genotoxic profile that compares the survival fraction of mutant ES cells to their parental controls at 10% (black bar) and at 1% (grey bar) cell survival **A.** Exposure to 1,4-Benzoquinone (BQ). **B.** Exposure to etoposide (ETO). **C.** Exposure to camptothecin (CPT).

The mutant cells used for the BQ genotoxic profile are summarized in Table 1. The survival difference between mutant relative to control cells is shown at BQ concentrations that reduce mutant cell survival by 90% and 99% (black and grey bars, respectively). For example, at a BQ dose that reduces the survival of *Brca1*-mutant cells by 90% and 99%; the mutant cells exhibited an 8-fold and a 17-fold increase in sensitivity (measured as reduced cell survival) compared to control cells, respectively. This observation suggests BQ induces DNA breaks and destabilizes replication forks since BRCA1 is needed to address these problems as a member of HR. In support, cells mutated for other DSB repair and replication fork maintenance genes also exhibited >5-fold hypersensitivity to BQ (Figure 1A). These include cells defective for FA (*Fancb*), nonhomologous end joining (NHEJ, *Ku70*) and interstrand crosslink repair (ICLR)/HR (*Ercc1*). By comparison, cells with a mutation in a lesion bypass gene, *Trex2*, caused BQ-resistance supporting the notion that DSB repair and replication fork maintenance are important for correcting BQ-induced lesions since *Trex2*-deletion is known to increase HR and NHEJ [23, 24]. The *Brca2*-mutant cells did not exhibit profound hypersensitivity even though BRCA2 is important for DSB repair and replication fork maintenance [25]. However, these cells produce wild type levels of a C-terminally truncated protein that is defective in RAD51 filament stability which causes only a minor phenotype [25–28]. Taken together, these data suggest that DSB repair and replication fork maintenance are essential for efficiently repairing BQ-induced damage.

Others have shown that BQ inhibits type II topoisomerases [29], leading to the formation of apoptosis-inducing type I topoisomerase cleavage complexes [30]. We therefore hypothesized that the genotoxic profile of BQ could resemble that of a type II topoisomerase poison such as etoposide (ETO) and potentially that of a type 1 topoisomerase inhibitor such as camptothecin (CPT). Indeed, like BQ, ETO exposure caused hypersensitivity in cells defective in DSB repair and replication fork maintenance (Figure 1B). Curiously, *Msh2^−/−^* cells exposed to ETO show antithetical responses depending on dose. It is possible at the lower dose *Msh2^−/−^* cells were resistant because MSH2 hindered replication fork progression while at the higher dose MSH2 were hypersensitive because MSH2 was needed to corrected damage that severely disabled replication or induced apoptosis. We then assessed the dose response to CPT in the mutant ES cell lines most sensitive to BQ and ETO (cells mutated for *Brca1*, *H2ax*, *Ku70* and *Ercc1*). Cells defective for HR (*Brca1*, *H2ax*) and ICLR (*Ercc1*) were hypersensitive to CPT, similar to BQ and ETO. Yet the NHEJ-defective cells (*Ku70*) were mildly resistant to CPT, suggesting that NHEJ performs a toxic function when encountering CPT-induced damage, unlike the response to BQ- and ETO-induced lesions. Thus, DSB repair and replication fork maintenance appear to be critical for correcting lesions caused by BQ, ETO and CPT.

### BQ causes less chromosomal damage than ETO and CPT

**Figure 2:**
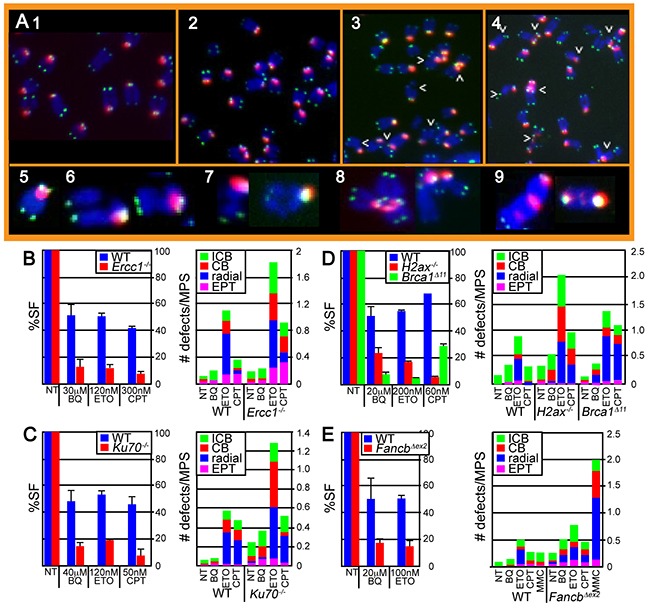
Evaluation of chromosome damage in metaphase spreads (MPS) after ES cells were exposed to BQ, ETO and CPT **A.** Images of *H2ax^−/−^* cells exposed to 1) nothing, 2) BQ, 3) CPT and 4) ETO. Arrowheads point to chromosomal abnormalities. Enlarged representative examples of chromosomes include 5) normal, 6) isochromatid break (ICB), 7) chromatid break (CB), 8) radial and 9) extrapericentromeres and telomeres (EPT). **B–E.** The survival fraction (%SF) is shown on the left panel and the # of chromosomal defects is shown on the right panel. (B) Wild type and *Ercc1*-mutant IB10 cells. (C) Wild type and *Ku70*-mutant J1 cells. (**D**) Wild type and *H2ax*- and *Brca1*-mutated TC1 cells. (E) Wild type and *Fancb*-mutated AB2.2 cells. Cells were also exposed to an equivalently toxic dose of Mitomycin C (MMC), a crosslinking agent that is known to be very toxic to FA-defective cells. The concentration for CPT (100 nM, 16 hours) and MMC (30 nM, 16 hours) results in ~ 10% and 90% survival for control cells and ~ 10% and <0.001% survival for *Fancb^Δex2^* cells as previously reported [38]. Note that MMC induces a much larger level of cell death and radials relative to control cells than the other agents relative to control cells demonstrating that *Fancb^Δex2^* cells are particularly susceptible to MMC as compared to the other agents. The total number of MPS observed for each bar and statistics are shown in supplemental tables 1 and 2, respectively.

The BQ genotoxic profile predicts that cells defective in DSB repair and replication fork maintenance will exhibit extensive chromosomal damage after BQ exposure; therefore, we evaluated chromosome integrity of relevant ES cell lines using two-color fluorescence in situ hybridization (FISH) on metaphase spreads (MPSs). DAPI counterstained chromosomes were stained with a telomere and a pericentromere probe [31]. We scored isochromatid breaks, chromatid breaks, radials and EPTs (extra pericentromeres and telomeres) (Figure 2A) [32]. Chromatid breaks indicate one-ended breaks observed at collapsed replication forks while isochromatid breaks indicate failed strand exchange intermediates that break both chromatids. Radials suggest chromosomal structures that result from the fusion of multiple chromosomes, such as those commonly observed in cells derived from FA patients after exposure to DNA crosslinking agents [33]. EPTs are complex rearrangements that occur in HR-defective cells that implicate multiple fusions possibly due to faulty replication or the imprecise joining of multiple DSBs [32]. Thus, two-color FISH will detect a range of chromosomal defects.

BQ-induced damage was compared to that of ETO and CPT because all three genotoxins showed similar results in the screening assay. We focused on those mutant ES cells that showed a hypersensitive phenotype in response to BQ (*Ercc1^−/−^*, *Ku70^−/−^*, *H2ax^−/−^*, *Brca1^Δ11/Δ11^* and *Fancb^Δex2^* ES cells). Physiologically comparable doses of genotoxins were used that produced a survival fraction of ~40-70% and ~5-20% for control and mutant cells, respectively (Figure 2B–2E, left panels). Therefore, these genotoxins are being compared at comparably toxic doses. At these doses, BQ caused fewer chromosomal defects than either ETO or CPT (Figure 2B–2E, right panels, Supplementary Table S1A-S1D, Supplementary Table S2A-S2D). Yet, this difference dissipates in *Fancb^Δex2^* cells implicating the FA pathway as central for repairing BQ-induced damage. FANCB is an essential member of the FA core complex [34] that is capable of monoubiquitinating FANCD2 in a minimal subcomplex [35] and its disruption completely destroys core complex function [36]. The FA pathway is important for replication fork maintenance, in particular protection of the nascent strand [37, 38]. These results suggest that BQ-mediated DNA damage has the potential to disrupt replication.

### BQ is more efficient at stalling replication forks than ETO and CPT

**Figure 3:**
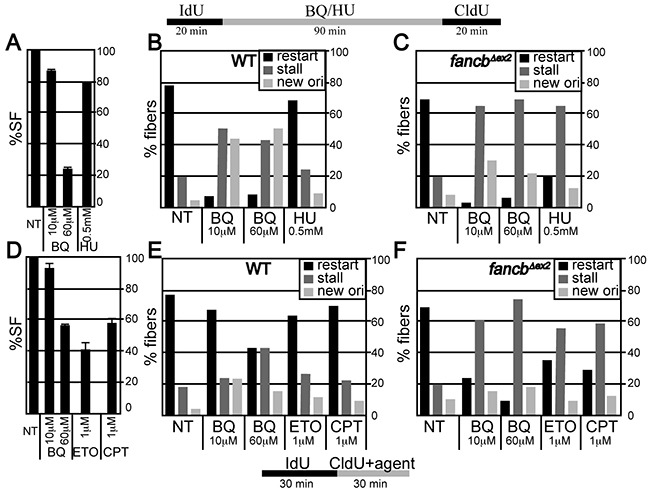
Fiber analysis **A-C.** ES cells were exposed to IdU for 20 minutes and then agent (BQ or HU) for 90 minutes and then CldU for 20 minutes. (A) Percent survival fraction (%SF) using the identical condition as the fiber analysis. (B) Fiber analysis in wild type AB2.2 cells. (C) Fiber analysis in *Fancb*-mutant cells. **D-E.** ES cells were exposed to IdU for 30 minutes and CldU + agent for 30 minutes. (D) Percent survival fraction (%SF) using the identical conditions for the fiber analysis. **E.** Fiber analysis in wild type AB2.2 cells. **F.** Fiber analysis in *Fancb*-mutant cells. The total number of fibers observed for each bar and statistics are shown in supplemental tables S3 and S4, respectively.

DNA fiber analysis was used to measure replication fork restart in response to BQ in control and *Fancb^Δex2^* cells since the FA pathway is important for coping with BQ-induced DNA damage and since FA stabilizes replication forks [37, 38]. In this assay, BQ was compared to a positive control, hydroxyurea (HU). HU inhibits ribonucleotide reductase, which depletes nucleotides [39] to impair the restart of replication forks [25]. Physiologically comparable doses of genotoxins (1.5 hr of 10 μM BQ or 0.5 mM HU) were used that produced a survival fraction of ~80-90% in control cells (Figure 3A). This particular dose of HU does not cause breaks [23, 25] and has a mild effect on replication fork restart and origin firing in control cells (Figure 3B, Supplementary Tables S3 & S4). In contrast, BQ significantly reduced levels of restart while it increased levels of new origin firing (Figure 3B). In *Fancb^Δex2^* cells, both BQ and HU reduced restart and new origins, with BQ being more severe (Figure 3C). A higher BQ concentration did not exacerbate these observations, suggesting the lower dose already produced the maximal effect (Figure 3B, 3C).

BQ's impact on replication fork restart was compared to that of ETO and CPT, in control and *Fancb^Δex2^* cells. Physiologically comparable doses of genotoxin (0.5 hr of 60 μM BQ or 1 μM ETO/CPT) were used, producing a survival fraction of ~40-60 % for control cells (Figure 3D). In control cells, BQ reduced replication fork restart more than ETO and CPT (Figure 3E). By contrast in *Fancb^Δex2^* cells, all three genotoxins reduced replication fork restart with BQ being the most severe (Figure 3F). Thus, BQ blocks replication fork restart more than HU, ETO and CPT, which is exacerbated in FA-defective cells.

### BQ is not efficient at inducing ATR/DNA-PK_CS_ responses to stalled replication forks

**Figure 4:**
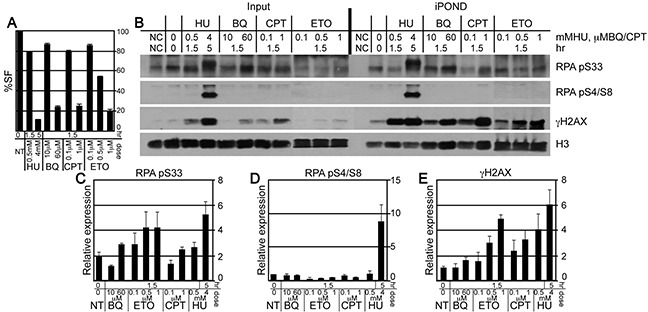
The purification of γH2AX and RPA at the nascent replication strand using iPOND **A.** The percent survival fraction (%SF) using the same condition as for iPOND. **B.** Western blot to evaluate the protein concentrations at purified nascent replication strands. **C-E.** Graphs that depict the quantitation of 3 Western blots for (C) γH2AX, (D) RPA pS33 and (E) RPA pS4/S8. Error bars are shown for the average of 3 experiments.

A fork that fails to restart can be temporarily stalled or more severely collapsed, possibly without a replisome [40]. The latter is more likely to form an intermediate structure amenable to a chromosomal rearrangement with potential for disease development, yet they are indistinguishable at the level of fiber analysis. Therefore, iPOND was used to observe the severity of the defect in replication fork restart by observing the phosphorylation pattern of RPA 32 [32, 41]. RPA 32 associates with single strand DNA at replication forks [42]. ATR phosphorylates serine 33 for a mild response while DNA-PK_CS_ phosphorylates serines 4 and 8 for a severe response [43]. For ES cells, a low HU dose (0.5 mM, 1.5 hours) induces an ATR response while a high HU dose (4 mM, 5 hours) induces both an ATR and a DNA-PK_CS_ response [25]. Previously we showed that the high HU dose produced more chromatid breaks; thus indicating collapsed forks with DSBs [25]. We compared similar physiologically toxic doses of BQ, ETO and CPT to that of low and high dose HU (Figure 4A). We found that all genotoxins caused an ATR response at the high doses but only ETO and HU caused this response at the low doses (Figure 4B, 4C). Furthermore, only high dose HU caused a DNA-PK_CS_ response (Figure 4B, 4D). For confirmation, we also purified γH2AX since it recognizes DNA DSBs [44] and single strand DNA at stalled replication forks [41]. We found that γH2AX levels directly correlated with the severity of the response such that the highest levels purified in HU exposed cells followed by ETO, CPT and BQ. Thus, the purification of γH2AX correlates with dose severity similar to RPA 32 phosphorylation. Therefore, from the genotoxins tested here, BQ is efficient at inhibiting fork restart (fiber analysis), but not efficient at inducing an ATR/DNA-PK_CS_ response or a γH2AX response (iPOND).

### BQ causes stalled replication forks to regress

**Figure 5:**
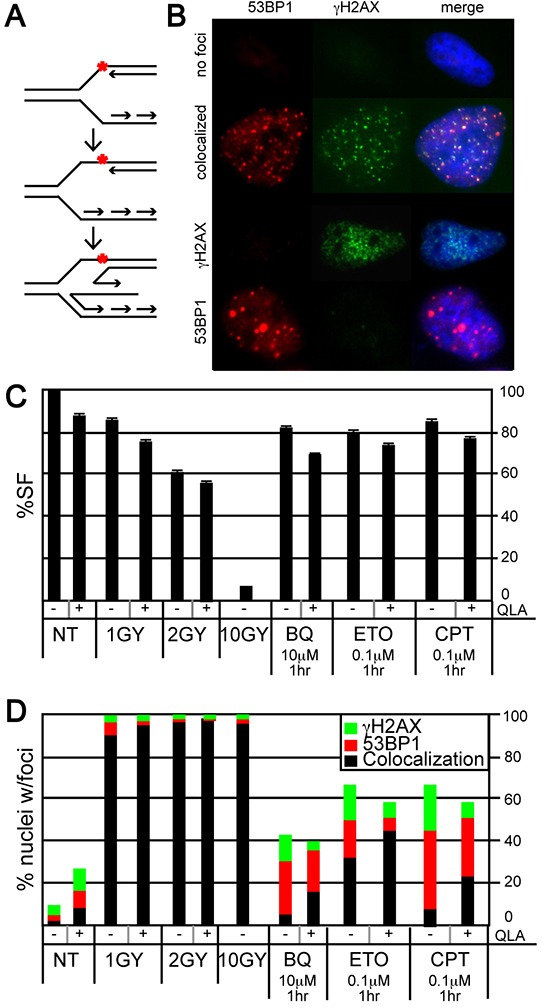
Evaluation of γH2AX and 53BP1 foci in HeLa cells exposed to BQ **A.** The formation of a regressed fork (chicken foot). The red asterix is a symbol for DNA damage that stalls a fork. This damage could be a CPT-type 1 topoisomerase cleavage complex. **B.** Representative examples of nuclei with no foci, colocalized foci, γH2AX foci, and 53BP1 foci. **C.** Survival fraction after exposure to ionizing γ-radiation [IR: 1-10 Gray (Gy)], olaparib (OLA, 10 μM), BQ, ETO and CPT. **D.** The percentage of nuclei with separated or colocalized γH2AX and 53BP1 foci. Ten or more foci are needed to be positive. The total number of nuclei observed for each bar and statistics are shown in supplementary tables S5 and S6, respectively.

We next observed the nature of individual stalled forks and DSBs induced by BQ since fibers suggest BQ efficiently inhibits fork restart but iPOND suggests minimal ATR/DNA-PK_CS_ responses indicating that BQ-induced stalled forks are stabilized and not subject to these responses. Previously, CPT was shown to induce stalled forks that regress and form a chicken foot (Figure 5A). Fork regression could stabilize stalled forks to minimize ATR/DNA-PK_CS_ responses. The localization of γH2AX and 53BP1 foci can be used to identify regressed forks. Similar to γH2AX, 53BP1 associates with damaged DNA to form nuclear foci [45]. Nuclei without foci implicate little to no damage (Figure 5B, 1^st^ row). Nuclei with colocalized foci (a merge of γH2AX and 53BP1) implicate replication-independent damage that does not cause chicken feet as seen after exposure to γ-radiation (Figure 5B, 2^nd^ row) [46]. By contrast nuclei with single-protein foci (either γH2AX or 53BP1) implicate replication-dependent damage that cause chicken feet as seen after exposure to CPT (Figure 5B, 3^rd^ and 4^th^ rows). Furthermore, PARP1 stabilizes chicken feet by inhibiting RECQ1 helicase and the PARP1 inhibitor, olaparib (OLA) reduces chicken feet [47, 48] such that exposure to OLA will reduce the number of nuclei with single-protein foci and an increase of nuclei with merged foci [47, 48]. Thus, the analysis of γH2AX and 53BP1 foci will measure the presence of PARP1-stabilized chicken feet.

We evaluated γH2AX and 53BP1 foci to determine if BQ induced PAPR1-stabilized chicken feet. Gamma-radiation and CPT were used as controls since both cause DSBs but only CPT promotes chicken feet. Cells were exposed to a variety of γ-radiation doses that result in a survival fraction of ~97% (1Gy), ~60% (2 Gy) and ~7% (10 Gy). A dose of genotoxin that is physiologically comparable to the milder γ-radiation doses was used for the other genotoxins (Figure 5C). In addition, a mild OLA dose was used that had only minimal or no impact on cell survival in the presence of genotoxin (Figure 5C). As expected, γ-radiation (with or without OLA) resulted in a strong majority of nuclei with colocalized foci (no chicken feet) while CPT resulted in a majority of nuclei with single-protein foci (chicken feet) and OLA reduced the proportion of these nuclei (implicating PARP1-stabilized regressed forks) (Figure 5D, Supplementary Tables S5 & S6). Exposure to BQ was almost identical to CPT (except BQ-exposed cells had fewer nuclei with foci) while exposure to ETO was intermediate to γ-radiation and CPT. Thus, BQ appears to cause PARP1-stabilized chicken feet much like CPT, suggesting a similar mechanism of action to this type 1 topoisomerase inhibitor.

### BQ inhibits the function of topoisomerase 1

**Figure 6:**
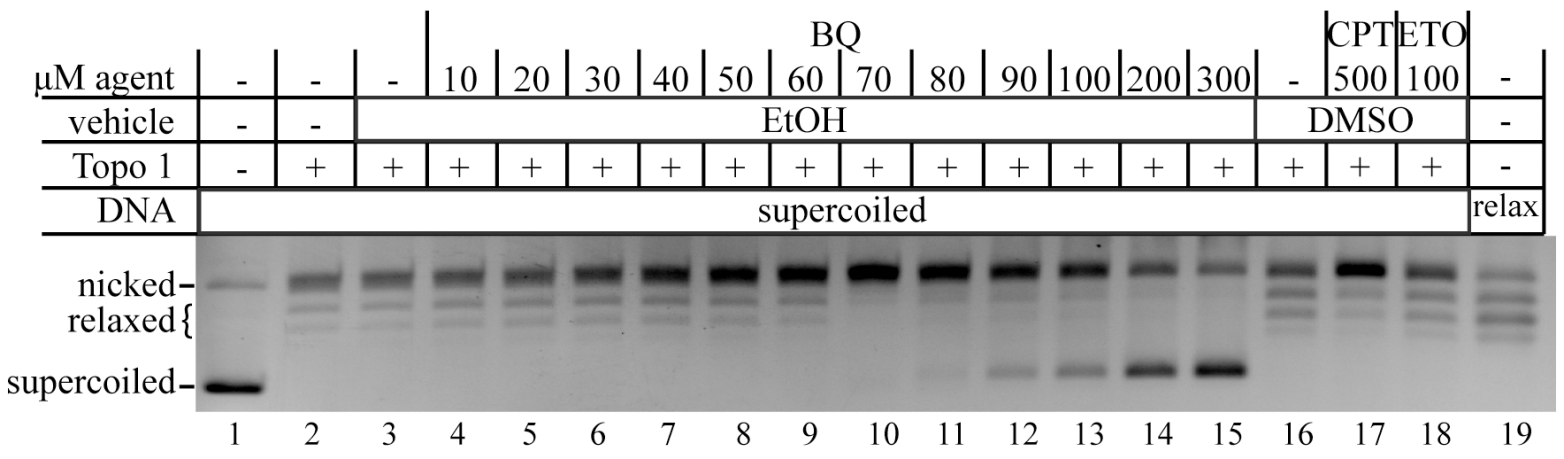
BQ inhibits type 1 topoisomerase (topo 1) CPT is a positive control and ETO is a negative control. The relaxed DNA shown in lane 19 is a control that came with the kit.

The γH2AX and 53BP1 foci analysis supports the possibility that BQ directly inhibits type 1 topoisomerases. To test this notion, we used a standard biochemical assay that measures nicking and relaxing of a supercoiled DNA substrate. ETO (100 μM), a type 2 topoisomerase inhibitor, served as a negative control and did not nick or relax the supercoiled substrate, while CPT (500 μM) served as a positive control and indeed inhibited the relaxation of nicked circular DNA (Figure 6). Similar to CPT, BQ progressively inhibited the relaxing of nicked circular DNA from 20-70 μM and progressively inhibited the nicking of supercoiled DNA from 70-300 μM. Thus, BQ directly interferes with topoisomerase I activity.

## DISCUSSION

Here we explore the nature of BQ genotoxicity since it is the primary metabolite suspected to cause the hematopoietic damage observed in people exposed to benzene. A non-biased approach was taken in mouse ES cells to identify the most critical pathways that address BQ-induced DNA damage. We found that DSB repair and replication fork maintenance pathways were essential for addressing these lesions. Moreover, we discovered that BQ interfered with type 1 topoisomerase which is consistent with a pathway necessary to maintain cell survival, replication fork stability and genome integrity.

For this proposal we performed our screen in mouse ES cells and comparisons to other cell types should be made with an understanding of their differences and similarities. One difference from many cells is that p53 exhibits some, but not all, its functions. Specifically, ES cells do not exhibit a p53/p21-mediated G_1_/S checkpoint even though they exhibit certain hallmarks like an IR-induced ATM/ATR response and p53-mediated increase in p21 transcription. In spite of these characteristics, there is no increase in p21 protein due to epigenetic regulation and proteasome-mediated degradation [49]. This is likely to prevent differentiation [50]. However, this p53-mediated response does not seem to be important for suppressing cancer since mice defective for it, but not other p53 responses (*p53^3KR/3KR^*) [51] and mice deleted for p53 DNA damage targets (*p21^−/−^*, *Puma^−/−^*, *Noxa^−/−^*) [52] do not exhibit early lymphomas and sarcomas as do *p53*-null mice [53]. In addition, there are intra S- and G_2_ checkpoints that are independent of p53 [54]. Moreover, human ES cells commit to apoptosis instead of checkpoint activation when exposed to DNA replication inhibitors [55] and our data concurs for mouse ES cells [38]. These qualities should be understood when using mouse ES cells in order to fairly compare these cells to other cell types like cancer cells and hematopoietic stem cells (HSCs).

There are similarities between mouse ES cells to cancer cells and HSCs. Like ES cells, cancer cells are often mutant for p53 (hence no G_1_/S checkpoint) [56] with elevated glycolysis (Warburg effect) [57–60]. They both are also pluripotent, immortal and oncogenic [61]. ES cells like stem cells exhibit self-renew and can be programmed to differentiate [62]. ES cells are also similar to HSCs with regard to the diminished importance of the p21 response. In mouse HSCs, p21 is not essential for steady-state hematopoiesis (but could be important under conditions of IR-induced stress) [63, 64]. Relevant for this project, mouse ES proliferate rapidly and are endowed with strong replication fork maintenance properties. This is important for studying toxins that impact HSCs since replicative stress is a major contributor to their functional decline and since HSCs accumulate DNA damage as they leave a quiescent state as a direct consequence of replicative stress [65, 66]. In addition, defects in pathways that suppress broken replication forks lead to a collapse of the hematopoietic system when challenged [67]. In concurrence with these observations, we find in a nonbiased screen with ES cells that DSB repair and replication fork maintenance pathways are essential to address BQ-induced damage. Of note, mouse ES cells mutated for excision repair genes display an obvious phenotype; therefore, the absence of phenotype for these mutant cells exposed to BQ is not due to naturally diminished excision repair. Thus, BQ likely induces replicative stress that leads to DSBs to cause hematopoietic toxicity.

We propose the following model to explain benzene-induced hematopoietic toxicity. The benzene metabolite, BQ suppresses type 1 topoisomerases to inhibit replication fork restart and increase supercoiling upstream of the fork. Then PARP1-stabilized fork regression ameliorates the tension caused by supercoiling and minimizes the ATR and DNA-PK_CS_ responses to phosphorylate RPA 32. An interesting observation is that BQ causes fewer chromosomal anomalies than either ETO or CPT at similarly toxic doses based on cell survival. It is possible that BQ is less mutagenic than ETO or CPT since it can inhibit type 1 topoisomerase nicking that would otherwise generate substrates for joining. Yet, imperfect repair or faulty maintenance of the fork would still lead to chromosomal rearrangements with the potential to develop into a hematopoietic cancer. This model proposes that people with poor genome maintenance capacity are at high risk for BQ-induced disease; of particular importance is their ability to repair DNA DSBs and maintain stabile replication forks. Our results are in concordance with reports that describe defects in HR and FA predispose people to hematopoietic cancers like MDS and AML [16, 68–70]. These individuals would likely be more susceptible to BQ toxicity further increasing their risk to develop hematopoietic disease. Furthermore, our results correspond to reports that show chemotherapeutics like ETO cause therapy-related MDS and AML (t-MDS/AML) [71, 72]. Benzene pollution would also have a greater impact on cancer patients. For such people, locating to a low-benzene environment would reduce their risk of t-MDS/AML.

## MATERIALS AND METHODS

### Mutant cell lines

For the experiment shown in Figure 1, we used ES cells mutated for NER (*Xpa* [73], *Xpc* [74]) MMR (*Msh2* [75]), error-free postreplication repair (*Rad18* [76]), FA (*Fancb* [77]) and nonhomologous end joining (*Ku70* [78]). Complete ablation of HR is cell lethal [79]; therefore, we use null cells for several genes that contribute to, but are not essential for HR (*H2ax* [80], *Rad52* [81], *Rad54* [82]). In addition, we have cells that are partially defective for essential proteins that include a deletion of *Brca2* exon 27 [26] and deletion of *Brca1* exon 11 [83]. We also use cells defective for HR regulation that include mutations in the helicases *Blm* [84] and *Recql5* [85]. We also have cells defective for the endonucleases *Mus81* [86] and *Ercc1* [87] and the exonuclease *Trex2* [88]. All mutants were compared to their parental clone as we previously described [21].

### Cell culture conditions

Mouse embryonic stem (ES) cells were cultured in Hyclone Dulbecco's high glucose Modified Eagles Medium (GE Healthcare) with 15% fetal bovine serum (FBS) (Gemini bio-products), 2 mM glutamine (GIBCO), 30 μg/mL penicillin (Sigma), 50 μg/mL streptomycin (GIBCO), 10^−4^ M β-mercaptoethanol (Sigma) and 1000 units/mL leukemia inhibitory factor (Gemini bio-products). Mouse ES cells were cultured on cell culture dishes (Corning) coated with 0.1% gelatin. HeLa cells were maintained in Minimal Eagle Medium (GIBCO) with 10% FBS, 2 mM glutamine, 30 μg/mL penicillin and 50 μg/mL streptomycin. All cell lines were grown at 37°C in a 5% CO_2_ humidified incubator.

### Dose response curves

The dose response curves were performed with a variety of mutant cells as described [21] [22]. BQ was suspended in ethanol.

### Two-color fluorescent *in situ* hybridization (FISH)

Mouse ES cells were cultured on 10 cm plates and treated with BQ, CPT, ETO or MMC at the doses described in figure 2. The remainder of the experiments were performed as described elsewhere [77].

### DNA fiber assay

For the experiment shown in Figure 3A-3C, mouse ES cells (AB2.2 and *Fancb^Δex2^*) were cultured on a 6-well plate (Corning) and labeled with 25 μM 5-Iodo-2′-deoxyuridine (IdU) (Sigma) at 37°C for 20 minutes. Labeled cells were washed twice with fresh media and treated 1.5 hours with either BQ (10 μM or 60 μM) or HU (0.5 μM). Cells were again washed twice with fresh media and then labeled with 250 μM 5-Chloro-2′-deoxyuridine (CldU) at 37°C for 20 minutes. The remainder of the experiment was performed as described [25]. For the experiment shown in Figure 3D-3F, cells were labeled with IdU as described above. Labeled cells were washed twice with fresh media and then treated for 30 minutes with fresh media containing 250 μM CldU plus BQ (10 μM or 60 μM) or ETO (1 μM) or CPT (1 μM). The remainder experiments were performed as previously described [25].

### Isolation of proteins on nascent DNA (iPOND)

For the experiment shown in Figure 4, mouse ES cells (AB2.2) were expanded on 15 cm plates. Cells were incubated with 10 μM 5′-ethynyl-2′-deoxyuridine (EdU) (Invitrogen) for 15 minutes. Cells incorporated with EdU were washed with fresh media and treated with agents BQ (10 μM for 1.5 hours or 60 μM for 1.5 hours), ETO (0.1 μM for 1.5 hours or 0.5 μM for 1.5 hours or 1 μM for 1.5 hours), CPT (0.1 μM for 1.5 hours or 1 μM for 1.5 hours) or HU (0.5 mM for 1.5 hours or 4 mM for 5 hours). The remainder of the experiment was performed as described [25].

### Immunofluorescence (foci analysis)

For the experiment shown in Figure 5, HeLa cells were plated on LabTek chamber slides (Thermo scientific) and treated with BQ or ETO or CPT or γ-radiation (Mark1 gamma radiation source from Shepard and Associates) with or without olaparib (Sellekchem) at the doses shown in the figure. Cells were rinsed with PBS and fixed in 2% formaldehyde at room temperature for 10 minutes. Cells were then rinsed with PBS and permeabilized with 0.5% Triton X-100 at room temperature for 10 minutes. After washing with PBS for 5 minutes, three times, cells were blocked in 4% non-fat milk in PBS at room temperature for 1 hour followed by 4°C overnight incubation with a rabbit polyclonal antibody directed against 53BP1 (1:250) (A300-273A, Bethyl laboratories) and mouse monoclonal antibody directed against γH2AX (1:250). The next morning cells were washed with PBS three times and incubated with Alexa Fluor 594-conjugated goat anti-rabbit IgG and Alexa Fluor 488-conjugated goat anti-mouse IgG (both 1:1000, Molecular Probes) at RT for 1 h. After three more washes with PBS, cells were washed with PBS three times and mounted in Vectashield (Vector laboratories). Images were captured by Axioplan2 and analyzed by AxioVision software. A cell was scored as positive if it contained ≥10 foci. More than 350 cells per each sample were counted and the counting assessment was done blind.

### DNA cleavage assay with topoisomerase 1

DNA cleavage assays (n=3) were performed with the Topoisomerase I assay kit from Topogen. Reaction mixtures contained 1X TGS buffer (100 mM Tris (pH 7.9), 10 mM EDTA, 1.5 M NaCl, 1% BSA, 1 mM spermidine, 50% glycerol) and 125 ng/μl supercoiled or relaxed pHOT-1 DNA. p-BQ (10-300 μM), CPT (100-500 μM) as a positive control or 100 μM ETO as a negative control and 10 U recombinant human topoisomerase I were added last and incubated at 37°C for 1 hour. Reactions were stopped by addition of 10% SDS. Next, 5 ng/μl proteinase K was added to detect clear DNA cleavages and reactions were incubated at 37°C for 30 minutes. Reactions were terminated with addition of 1X gel loading buffer (5% sarkosyl, 0.125% bromophenol blue, 25% glycerol). Samples were electrophoresed onto a 1% agarose gel (Sigma) at 55 V for 3 hours. The gel was stained with 0.5 μg/ml ethidium bromide (Fisher biotech) in 1X TAE buffer (40 mM Tris-Acetate and 1 mM EDTA (Ambion) for 30 min. followed by a 15 min. destaining in ddH_2_O. DNA bands were captured by Gel logic 200 imaging system (Kodak) and Carestream MI version 5.0.7.24 software (Carestream Health). This assay was performed three times with similar results.

## SUPPLEMENTARY TABLES


