# Supplementary material for: A mechanism for 1,4-Benzoquinone-induced genotoxicity
Source: Oncotarget. 2016 Jun 20;7(29):46433–47. doi: 10.18632/oncotarget.10184 (PMC5216808; doi:10.18632/oncotarget.10184)
Supplement: Supplementary file 1 [file oncotarget-07-46433-s001.pdf]

A mechanism for 1,4-Benzoquinone-induced genotoxicity

SUPPLEMENTARY TABLES

Supplementary Table S1: Total number of chromosomal abnormalities

(A)

Genotype	IB10				Ercc1			
**Treatment**	**NT**	**BQ**	**CPT**	**ETO**	**NT**	**BQ**	**CPT**	**ETO**
No. of MPS	262	137	126	113	221	117	150	106
No. of CB	6	0	5	19	8	5	38	46
No. of ICB	12	17	13	17	14	15	29	50
No. of radial	2	0	5	19	8	5	38	46
No. of EPT	9	7	19	15	13	6	45	23

(B)

Genotype	J1				Ku70			
**Treatment**	**NT**	**BQ**	**CPT**	**ETO**	**NT**	**BQ**	**CPT**	**ETO**
No. of MPS	286	296	120	135	249	269	120	116
No. of CB	3	7	11	19	12	27	4	57
No. of ICB	8	7	14	13	34	48	19	21
No. of radial	0	1	30	43	4	4	34	61
No. of EPT	1	2	1	1	8	13	3	9

(C)

Genotype	TC1				H2ax				Brca1			
**Treatment**	**NT**	**BQ**	**CPT**	**ETO**	**NT**	**BQ**	**CPT**	**ETO**	**NT**	**BQ**	**CPT**	**ETO**
No. of MPS	173	235	119	114	128	132	157	112	262	114	134	132
No. of CB	3	4	1	10	6	21	55	75	9	12	19	25
No. of ICB	23	63	36	36	27	39	39	66	10	13	25	38
No. of radial	0	0	7	43	1	5	47	82	4	14	91	106
No. of EPT	0	4	0	11	3	4	4	6	9	5	12	13

(D)

Genotype	AB2.2					fancB				
**Treatment**	**NT**	**BQ**	**CPT**	**ETO**	**MMC**	**NT**	**BQ**	**CPT**	**ETO**	**MMC**
No. of MPS	75	61	97	81	71	87	98	105	113	112
No. of CB	0	1	2	5	4	2	12	14	12	56
No. of ICB	6	7	9	12	10	12	13	13	34	18
No. of radial	0	0	2	20	1	3	14	11	24	128
No. of EPT	0	0	1	5	4	4	11	10	17	18

Supplementary Table S2: Statistics for chromosomal abnormalities

(A)

IB10	NT vs BQ	NT vs CPT	NT vs ETO
CB	0.078583752	0.158983932	7.49889E-07
ICB	0.007882828	0.053031542	0.00107523
Radial	0.430611705	0.075079209	1.48288E-45
EPT	0.58867548	8.10318E-05	0.000831625
**Ercc1**	**NT vs BQ**	**NT vs CPT**	**NT vs ETO**
CB	0.217745063	1.30056E-09	5.19516E-19
ICB	0.068554323	0.000239568	1.10452E-17
Radial	0.278210291	3.42802E-06	7.04401E-41
EPT	0.969541744	8.69345E-10	4.35048E-05
**IB10 vs Ercc1**	**BQ**	**CPT**	**ETO**
CB	0.01978593	2.49633E-06	3.25065E-05
ICB	0.927409195	0.056275194	5.46577E-07
Radial	1	0.003606379	0.697731127
EPT	0.780342949	0.005395324	0.142495651

(B)

J1	NT vs BQ	NT vs CPT	NT vs ETO
CB	0.124809265	0.000148106	7.85698E-08
ICB	0.946238946	0.000774106	0.005679447
Radial	0.508591065	9.59207E-18	4.15108E-23
EPT	0.382009272	0.417442073	0.436715304
**Ku70**	**NT vs BQ**	**NT vs CPT**	**NT vs ETO**
CB	0.037335116	0.70118699	3.22283E-23
ICB	0.236179832	0.688726795	0.342545085
Radial	0.273912922	3.84849E-12	1.17586E-31
EPT	0.477072395	0.251558583	0.09848477
**J1 vs Ku70**	**BQ**	**CPT**	**ETO**
CB	0.000259419	0.109598583	3.85877E-09
ICB	1.38745E-09	0.75618115	0.076576387
Radial	0.133969694	0.661454675	0.001392682
EPT	0.004988797	0.249986759	0.004512139

(C)

TC1	NT vs BQ	NT vs CPT	NT vs ETO
CB	0.296222632	0.340099452	0.011888498
ICB	0.001449663	0.00067951	0.000574851
Radial	1	0.001677034	8.55107E-18
EPT	0.108865932	1	2.86576E-05

(*Continued*)

TC1	NT vs BQ	NT vs CPT	NT vs ETO
**Brca1**	**NT vs BQ**	**NT vs CPT**	**NT vs ETO**
CB	0.012142301	0.407534247	0.055438263
ICB	0.009666723	2.1849E-06	2.75972E-12
Radial	2.37211E-05	1.00011E-47	5.71994E-60
EPT	0.202079533	0.03730404	0.017103196
**H2AX**	**NT vs BQ**	**NT vs CPT**	**NT vs ETO**
CB	0.005744635	1.30062E-09	9.94549E-24
ICB	0.115108977	1.90803E-09	0.444673675
Radial	0.097801022	1.74016E-10	1.3258E-29
EPT	0.280795902	0.295570885	0.130574137
**TC1 vs Brca1**	**BQ**	**CPT**	**ETO**
CB	0.000617864	0.000223106	0.036315702
ICB	0.001736695	0.044980964	0.851090364
Radial	8.92507E-08	1.87551E-23	2.32067E-11
EPT	0.095313137	0.002300234	0.870594832
**TC1 vs H2AX**	**BQ**	**CPT**	**ETO**
CB	6.75673E-07	7.70467E-12	5.99373E-19
ICB	0.659728293	0.387475239	3.57147E-05
Radial	0.00572981	1.32779E-06	1.46868E-06
EPT	0.197987449	0.102972601	0.331587017

(D)

AB2.2	NT vs BQ	NT vs CPT	NT vs ETO	NT vs MMC
CB	0.448529412	0.316605467	0.018543409	0.05349276
ICB	0.694788005	0.982304829	0.279999459	0.362140305
Radial	1	0.316605467	1.24689E-05	0.48630137
EPT	1	0.563953488	0.035503845	0.05349276
**FancB**	**NT vs BQ**	**NT vs CPT**	**NT vs ETO**	**NT vs MMC**
CB	0.02293605	0.007923109	0.044763082	6.57913E-13
ICB	0.911908969	0.940976437	0.026357194	0.805789674
Radial	0.021911205	0.112812404	0.000578768	4.87175E-59
EPT	0.168109541	0.303913677	0.031040671	0.019674598
**AB2.2 vs FancB**	**BQ**	**CPT**	**ETO**	**MMC**
CB	0.012303846	0.00406805	0.41064819	1.29176E-09
ICB	0.93221283	0.630405528	0.021701655	0.878294686
Radial	0.005055786	0.031721572	0.69472111	1.16608E-58
EPT	0.003879962	0.018908129	0.090609391	0.059780746

Fisher’s exact test

Yates-corrected Chi-Square Test

Supplementary Table S3: Total number of DNA fibers

Cell lines	Treatment	No. of fiber	Restart	Stall	New origin
AB2.2	NT	1139	880	213	46
	0.5 mM HU 1.5h	505	342	119	44
	10 uM BQ 1.5h	726	49	361	316
	60 uM BQ 1.5h	794	59	338	397
	1 uM CPT 0.5h	639	439	142	58
	1 uM ETO 0.5h	624	392	162	70
	10 uM BQ 0.5h	716	472	167	77
	60 uM BQ 0.5h	812	347	344	121
FancB	NT	757	522	159	76
	0.5 mM HU 1.5h	778	175	539	118
	10 uM BQ 1.5h	1269	41	517	247
	60 uM BQ 1.5h	635	53	438	144
	1 uM CPT 0.5h	865	246	511	108
	1 uM ETO 0.5h	874	306	490	78
	10 uM BQ 0.5h	873	207	534	132
	60 uM BQ 0.5h	846	71	624	151

Supplementary Table S4: Statistics for replication fork stalling

	NT vs 10 uM BQ 1.5h	NT vs 60 uM BQ 1.5h	NT vs 0.5 mM HU 1.5h	
AB2.2	3.71755E-45	4.9605E-30	0.027836187	
FancB	1.23652E-19	4.14111E-72	5.42418E-80	

	NT vs 10 uM BQ 0.5h	NT vs 60 uM BQ 0.5h	NT vs 1 uM ETO 0.5h	NT vs 1 uM CPT 0.5h
AB2.2	0.019136108	6.86093E-30	0.085329301	0.000462293
FancB	8.56433E-60	2.55562E-98	4.39149E-54	7.32663E-47

	10 uM BQ 1.5h	60 uM BQ 1.5h	0.5 mM HU 1.5h	
AB2.2 vs FancB	0.000121893	4.00033E-23	2.93729E-57	

	10 uM BQ 0.5h	60 uM BQ 0.5h	1 uM ETO 0.5h	1 uM CPT 0.5h
AB2.2 vs FancB	2.70718E-51	1.91101E-38	9.07954E-31	8.99874E-46

Yates-corrected Chi-square Test

Supplementary Table S5: The number of nuclei observed for the foci analysis

	Total	53BP1 (>10)	Colocalization	γH2AX (>10)
NT	904	52	17	64
10uM OLA 1h	737	126	57	134
1Gy+R1h	803	774	726	747
1Gy+10uM OLA	752	737	716	727
2Gy+R1h	459	453	450	455
2Gy+10uM OLA	531	524	520	529
10Gy+R1h	401	398	394	396
100nM CPT 1h	461	205	34	132
100nM CPT+10uM OLA 1h	874	443	189	243
10uM BQ 1h	569	175	23	84
10uM BQ+10uM OLA	617	215	86	107
100nM ETO 1h	365	178	109	167
100nM ETO+10uM OLA 1h	358	180	154	179

Supplementary Table S6: Statistics for foci

	53BP1 (>10)	Colocalization	γH2AX (>10)
NT vs 10uM OLA	3.57881E-13	2.63317E-08	1.10921E-11
NT vs 1Gy	2.3117E-305	4.803E-296	3.7449E-275
NT vs 1Gy+OLA	9.9153E-306	0	2.0774E-288
NT vs 2Gy	2.5789E-246	7.8832E-273	4.7623E-239
NT vs 2Gy+OLA	1.3461E-262	1.2076E-287	4.2648E-258
NT vs 10Gy	7.4943E-235	4.6317E-261	1.4717E-223
NT vs CPT	1.54659E-66	9.03282E-07	1.59977E-26
NT vs CPT+OLA	1.20359E-98	3.04284E-38	1.38875E-30
NT vs BQ	6.9055E-38	0.020305675	2.77615E-06
NT vs BQ+OLA	3.97421E-48	1.02867E-19	8.32935E-10
NT vs ETO	7.50394E-72	9.2611E-51	3.47663E-58
NT vs ETO+OLA	4.96131E-75	8.61584E-82	1.90288E-67

	Colocalization
1Gy vs 1Gy+OLA	0.000389048
2Gy vs 2Gy+OLA	0.917999024
CPT vs CPT+OLA	5.40415E-11
BQ vs BQ+OLA	6.90868E-09
ETO vs ETO+OLA	0.000320092

Yates-Corrected Chi-Square Test
